# Evolutionary morphology of the lizard chemosensory system

**DOI:** 10.1038/s41598-017-09415-7

**Published:** 2017-09-04

**Authors:** Simon Baeckens, Anthony Herrel, Chris Broeckhoven, Menelia Vasilopoulou-Kampitsi, Katleen Huyghe, Jana Goyens, Raoul Van Damme

**Affiliations:** 1 Laboratory of Functional Morphology, Department of Biology, University of Antwerp, Universiteitsplein 1, 2610 Wilrijk, Belgium; 2 UMR7179, CNRS/MNHN, 75005 Paris, France; 3 Department of Botany & Zoology, Stellenbosch University, Private Bag X1, Matieland, 7602 Stellenbosch, South Africa; 4 Present Address: Department of Organismic and Evolutionary Biology, Harvard University, Cambridge, Massachusetts USA

## Abstract

Foraging mode plays a pivotal role in traditional reconstructions of squamate evolution. Transitions between modes are said to spark concerted changes in the morphology, physiology, behaviour, and life history of lizards. With respect to their sensory systems, species that adopt a sit-and-wait strategy are thought to rely on visual cues primarily, while actively hunting species would predominantly use chemical information. The morphology of the tongue and the vomeronasal-organs is believed to mirror this dichotomy. Still, support for this idea of concerted evolution of the morphology of the lizard sensory system merely originates from studies comparing only a few, distantly related taxa that differ in many aspects of their biology besides foraging mode. Hence, we compared vomeronasal-lingual morphology among closely related lizard species (Lacertidae). Our findings show considerable interspecific variation indicating that the chemosensory system of lacertids has undergone substantial change over a short evolutionary time. Although our results imply independent evolution of tongue and vomeronasal-organ form, we find evidence for co-variation between sampler and sensor, hinting towards an ‘optimization’ for efficient chemoreception. Furthermore, our findings suggest species’ degree of investment in chemical signalling, and not foraging behaviour, as a leading factor driving the diversity in vomeronasal-lingual morphology among lacertid species.

## Introduction

Squamate reptiles (lizards and snakes) strongly rely on chemical cues to find food and to avoid predators, and many use chemical signals to communicate with hetero- or conspecifics. To perceive chemicals for the environment, squamates have evolved a highly sophisticated ‘vomeronasal-lingual’ system for chemoreception^1–5^.

Squamate ‘vomerolfaction’ is mediated by ‘tongue-flicking’ behaviour in which the tongue samples substrate-bound or air-born chemicals in the environment and delivers them to the paired vomeronasal organs (VNOs) above the roof of the mouth^6^. Unlike those of other vertebrates, the paired VNOs of squamates have lost their anatomical connection to the main olfactory system completely, and they operate as autonomous chemosensory organs^7–9^. Functionally, the main olfactory system and vomeronasal system are interrelated^10,11^. Volatile chemical stimuli are often first received through the nares and processed by the nasal organs (olfactory system), which subsequently triggers tongue-flick mediated vomerolfaction^2,12,13^. Specifically, vomerolfaction starts with the tongue delivering chemicals close to or into the vomeronasal openings. Subsequently, the molecules pass through the vomeronasal duct into the VNO’s lumen where they dissolve in the luminal fluid. The presence of the molecules in the fluid is detected by the sensory epithelium that lines the lumen dorsally, and consists of microvillous receptor neurons, sustentacular and basal cells^14^. Finally, the stimulated sensory epithelium relays information via the accessory olfactory nerves to the accessory olfactory bulbs of the telencephalon for processing^12,15,16^.

Although all squamates are equipped with this dual chemosensory apparatus, the degree of morphological specialisation of the vomeronasal-lingual system varies tremendously among higher squamate taxa, putatively reflecting the extent to which squamates utilize their chemosensory system^5,17^. In traditional accounts of the evolutionary history of lizards and snakes, the functional morphology of the tongue has played a key role^1,4,18–21^. Families of lizards have long been assigned to either the more ‘primitive’ Iguania—a group of sit-and-wait foragers, with fleshy tongues, and limited chemoreceptive abilities—or the ‘derived’ Scleroglossa that forage actively, use their jaws to capture food, and have a highly forked tongue for vomerolfaction^1,12,13,19,22–25^. Because a proficient chemosensory system can also be used in chemical communication, active foraging species are also thought to invest more in chemical signals^5^. Many ‘chemically-mediated’ lizards are, indeed, known to carry specialised cloacal or epidermal glands that play an important role in inter- and intraspecific chemical communication^26,27^. The idea that many aspects of the morphology, physiology, behaviour and life history of lizards evolve in concert to fulfil requirements associated with foraging mode derives support from comparisons of higher-level taxa^1,5,17,21^. For example, Schwenk^19^ observed a strong relationship between the degree of tongue forkedness and foraging mode, with lizard families that carry highly forked tongues spending more time actively searching for food. A deeply forked tongue is hypothesized to permit efficient prey-searching by *tropotaxis*; that is the ability to sample and sense relative signal strength from each side of the body separately and simultaneously^19^. These strongly bifid tongues are also often highly elongated, which is believed to increase the protrusibility and flexibility of the tongue^3^.

Apparently in line with ideas of economic design (‘symmorphosis’^28^), investment into the respective elements of the vomeronasal-lingual apparatus is also positively correlated. Taxa that have elongated, strongly bifid tongues (specialized for vomerolfaction) also have large VNOs, with large mushroom bodies and sensory-rich vomeronasal epithelia. They also tongue-flick at higher rates and sample larger volumes of air while doing so^5,17,29^.

Still, most of these findings on concerted evolution, driven by the specific requirements of the two foraging modes, stem from comparative analyses among a few, higher-level taxa. Comparing traits among groups that are only distantly related always merits a certain note of caution, as they differ in many aspects of their biology besides their mode of foraging due to their long and disparate evolutionary history. Comparative studies focusing on closely related species that share many aspects of their general morphology and ecology, but vary in their degree of foraging behaviour, might eliminate (many) confounding factors and may shed light on the true impact of foraging mode on vomeronasal-lingual morphology. Moreover, it is still uncertain whether the use and reliance on chemical communication has had an impact on the evolution of the vomeronasal-lingual morphology in traditional groups of ‘active foragers’. It is not unlikely that variation in species’ reliance on and use of chemical communication has influenced variation in chemosensory specialization even further. One can hypothesize that active foragers relying strongly on chemical communication are equipped with a more specialized vomeronasal-lingual system for chemoreception than active foragers relying less on chemical communication.

Here, we test for interspecific co-variation between the lingual- and VNO system using phylogenetic comparative methods and by examining the morphology of the tongue and VNOs of lizard species of the family Lacertidae. First, we use micro-CT imaging for three-dimensional reconstructions and volumetric measurements of lizard VNOs, and use basic morphometrics to examine tongue shape. Second, we explore the relationships between species’ vomeronasal-lingual morphology and their foraging behaviour, and also their overall investment in chemical signalling. Lacertid lizards are strongly chemically-oriented and rely on chemical cues and signals for foraging^30–34^, predator recognition^35–37^ and mate assessment^38–41^. Yet, lacertids greatly differ in their level of foraging activity^42–44^, and their chemical signalling system design^27,45,46^. We expect that species that rely strongly on chemical cues when searching for prey, and species that invest strongly in the production of chemical signals will also be best equipped with a more advanced or better-developed vomeronasal-lingual system to receive these cues and signals (i.e. an elongated bifid tongue, and large VNOs with a thick sensory epithelium). Therefore, we predict interspecific variation in lacertid vomeronasal-lingual morphology, which can be (at least partly) explained by variation in species’ level of reliance on chemical cues for foraging and/or chemical signals for chemical communication.

## Results

### Variation in vomeronasal-lingual morphology

**Figure 1:**
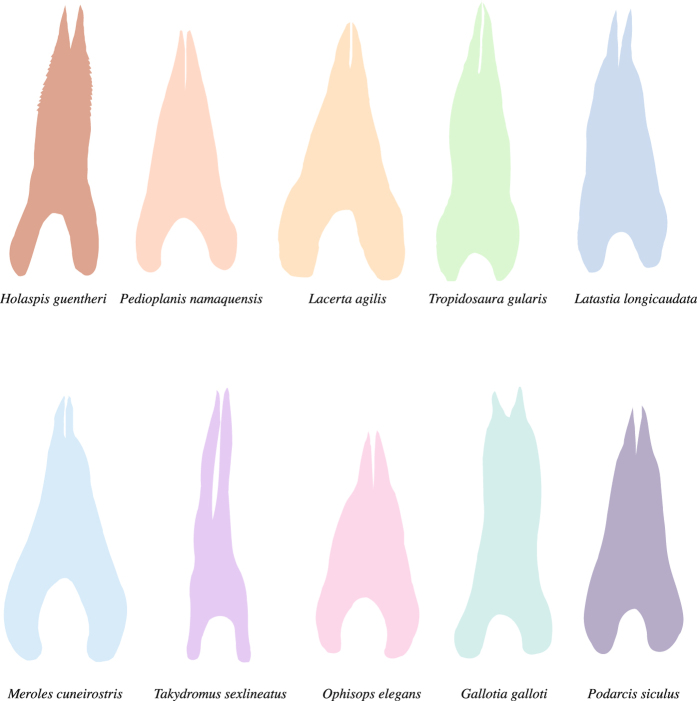
Illustration of the tongue form of ten lacertid lizards implemented in our study. All tongues are scaled to their head length (except *Takydromus*, which should be 43% smaller than visualized).

Morphometric analyses and micro-CT imaging revealed substantial disparity in vomeronasal-lingual morphology among lacertid species (e.g. Fig. 1). A summary of the VNO and tongue morphological measurements of the species used in this study is given in Table 1.

**Table 1:** Morphometrics of the tongue and vomeronasal organs of lacertid lizards, with additional data on proxies for chemical signalling investment and foraging behaviour activity.

Species	Head			Tongue									VNO				**Chemical signalling investment**			**Foraging activity**	
	**H**_**L**_	**H**_**W**_	**H**_**H**_	**T**_**area**_	**T**_**BW**_	**T**_**BL**_	**T**_**TL**_	**T**_**TW**_	**T**_**ML**_	**T**_**L**_	**T**_**elong**_	**T**_**FS**_	**VNO**_**sensvol**_	**VNO**_**senslum**_	**VNO**_**thick**_	**VNO**_**area**_	**FPN**	**OSP**	**TSP**	**MPM**	**PTM**
*Acanthodactylus boskianus*	16.43	9.71	7.97	24.55	4.05	3.05	2.28	1.74	5.17	10.49	1.28	1.31	0.2410	0.1597	0.2650	1.3447	26	0.027	0.702	2.01	28.20
*Acanthodactylus cantoris*	14.10	9.54	5.99	21.73	4.09	1.61	1.74	1.24	5.73	9.08	1.40	1.40	—	—	—	—	21	—	—	—	—
*Australocaerta australis*	15.76	10.45	6.40	27.57	3.70	0.95	3.09	1.91	7.67	11.71	2.07	1.62	—	—	—	—	18	0.072	1.296	—	—
*Dalmatolacerta oxycephala*	15.24	9.07	6.05	24.07	3.26	2.30	1.38	1.53	6.83	10.52	2.09	0.90	0.1785	0.0597	0.2430	0.9538	22	0.685	15.07	2.22	15.11
*Eremias acutirostris*	16.80	9.55	8.12	34.62	4.71	2.60	2.24	1.76	7.53	12.36	1.60	1.27	0.2481	0.0622	0.2130	1.2228	16	—	—	—	—
*Gallotia galloti*	21.46	12.87	10.95	54.58	5.10	2.58	1.99	2.07	11.67	16.24	2.29	0.96	0.3186	0.3022	0.1680	1.9713	27	0.016	0.432	—	—
*Holaspis guentheri*	10.80	7.28	3.62	11.35	2.17	1.84	1.61	1.36	4.71	8.16	2.17	1.18	0.0602	0.0143	0.1740	0.2669	20	0.256	5.120	—	—
*Ichnotropis capensis*	11.45	7.59	5.50	—	—	—	—	—	—	—	—	—	0.1465	0.0308	0.1760	0.6773	11	0.068	0.748	—	—
*Lacerta agilis*	17.96	12.19	9.78	44.45	6.36	2.26	2.43	1.71	8.22	12.91	1.29	1.42	0.2821	0.1180	0.1910	1.2163	13	—	—	0.21	1.59
*Lacerta schreiberi*	21.41	13.23	9.26	33.59	4.46	2.99	2.09	1.54	6.79	11.88	1.52	1.36	—	—	—	—	15	0,058	0.870	1.86	10.57
*Latastia longicaudata*	16.03	8.67	6.81	26.09	3.92	2.59	2.96	1.88	6.24	11.78	1.59	1.57	0.4045	0.1192	0.3310	1.1967	11	0.070	0.840	—	—
*Meroles cuneirostris*	14.22	9.66	6.92	25.61	4.30	3.12	2.01	1.19	5.42	10.54	1.26	1.69	—	—	—	—	21	—	—	—	—
*Meroles knoxii*	9.59	6.21	3.81	23.73	3.76	3.20	3.22	1.60	5.18	11.60	1.38	2.01	—	—	—	—	17	0.127	2,159	0.61	7.00
*Nucras tessellata*	14.07	9.71	7.14	23.44	3.90	2.29	1.92	1.60	5.47	9.68	1.40	1.20	—	—	—	—	13	0.049	0.637	2.90	50.20
*Ophisops elegans*	9.11	5.45	3.98	8.44	2.50	1.61	1.50	1.08	2.68	5.79	1.07	1.39	0.0692	0.0154	0.2070	0.3080	12	0.027	0.324	1.88	54.60
*Pedioplanis burchelli*	12.88	8.68	5.83	11.17	2.44	2.09	2.28	1.09	3.63	8.00	1.49	2.09	—	—	—	—	12	—	—	—	—
*Pedioplanis lineoocellata*	13.91	8.78	6.51	18.38	3.68	1.95	2.43	1.46	5.00	9.38	1.36	1.66	0.0998	0.0151	0.2680	0.3786	15	0.025	0.375	1.54	14.30
*Pedioplanis namaquensis*	10.11	5.85	3.90	10.53	2.46	1.73	1.98	1.20	3.59	7.30	1.46	1.65	—	—	—	—	13	—	—	1.87	54.00
*Phoenicolacerta laevis*	12.03	7.90	5.55	17.04	2.55	2.45	1.54	0.93	5.58	9.57	2.19	1.66	0.1146	0.0408	0.1890	0.7372	20	0.094	1.880	1.17	28.70
*Podarcis peloponnesiacus*	12.02	7.73	5.49	—	—	—	—	—	—	—	—	—	0.0946	0.0238	0.2213	0.4746	23	—	—	2.10	12.35
*Podarcis siculus*	17.46	11.16	7.52	33.38	4.87	1.84	2.52	1.87	7.74	12.10	1.59	1.35	0.1959	0.0884	0.2290	1.1216	21	0.174	3.654	—	—
*Psammodromus algirus*	14.82	9.06	6.77	27.88	3.63	2.59	1.67	2.31	6.97	11.23	1.92	0.72	0.2020	0.0899	0.1850	1.1097	17	—	—	2.95	20.68
*Takydromus sexlineatus*	14.18	6.99	5.61	4.65	1.36	0.79	3.05	0.81	2.42	6.26	1.78	3.77	0.1779	0.0449	0.2570	0.8362	2	0.362	0.724	1.60	13.80
*Timon lepidus*	49.19	38.24	23.29	182.43	13.19	4.17	4.80	3.41	15.43	24.41	1.17	1.41	—	—	—	—	14	0.222	3.108	—	—
*Tropidosaura gularis*	14.27	8.49	5.93	21.98	3.15	1.04	2.86	1.50	7.28	11.18	2.31	1.91	0.2530	0.0801	0.2270	1.1097	11	0.085	0.935	—	—
*Zootoca vivipara*	10.61	7.21	5.22	15.87	3.77	0.64	2.13	1.31	5.33	8.10	1.41	1.63	0.0908	0.0403	0.2880	0.5375	10	0.025	0.250	4.2	33.20

Abbreviations: H_L_ = head length, H_W _ = head width, H_H _ = head height, T_area_ = tongue surface area, T_BW_ = tongue base width, T_BL_ = tongue base length, T_TL _ = tongue tip length, T_TW = _tongue tip width, T_ML = _tongue mid length, T_L = _total tongue length, T_elong _ = tongue elongation, T_FS _ = tongue-fork score, VNO_sensvol_ = volume of VNO sensory epithelium, VNO_senslum_ = volume of VNO lumen, VNO_thick_ = thickness of VNO sensory epithelium, VNO_area_ = surface area of VNO sensory epithelium. All length measurements are noted in mm, area in mm^2^, and volumes in mm^3^. Average number of femoral pores (FPN), one-gland secretion production (OSP), total secretion production (TSP), foraging variables PTM (percentage time moving) and MPM (number of movements per minute).

Of all measures taken on the lizard tongues and VNOs, only T_FS_, T_elong_ and VNO_thick_ did not show a significant relationship with head length (see Table 2). All measures of tongue length (T_L_, T_BL_, T_ML_, T_TL_) and width (T_BW_, T_TW_) increased with head length, although somewhat less than expected under isometry. Tongue area scaled isometrically with head length, as did VNO_senslum_ and VNO_area_, whilst VNO_sensvol_ exhibited negative allometry.

**Table 2:** Relationships of tongue and vomeronasal organ (VNO) morphometrics with head size in lacertid lizards, obtained through phylogenetic generalised least square (pGLS) regressions. Slopes and intercepts of the confidence intervals (95%) are also presented.

Variables		Intercept	2.5% CI	97.5% CI	Slope	2.5% CI	97.5% CI	*r* ^2^	*P*
**With head length as independent**									
Tongue (n = 24)	T_area_	−0.66	−0.95	−0.38	1.73	1.49	2.01	0.71	<0.001
	T_BW_	−0.56	−0.76	−0.37	0.96	0.80	1.13	0.61	<0.001
	T_BL_	−0.39	−0.70	−0.08	0.59	0.33	0.85	0.19	0.033
	T_TL_	−0.28	−0.47	−0.10	0.50	0.35	0.65	0.33	0.003
	T_TW_	−0.58	−0.74	−0.41	0.65	0.51	0.79	0.51	<0.001
	T_ML_	−0.18	−0.37	0.020	0.84	0.69	1.00	0.57	<0.001
	T_L_	0.17	0.05	0.29	0.73	0.64	0.83	0.72	<0.001
	T_elong_	0.37	0.20	0.54	−0.09	−0.22	0.04	0.02	0.498
	T_FS_	0.20	−0.01	0.41	−0.11	−0.27	0.05	0.02	0.512
VNO (n = 17)	VNO_sensvol_	−3.16	−3.53	−2.80	2.06	1.75	2.38	0.74	<0.001
	VNO_senslum_	−5.17	−5.78	−4.56	3.40	2.90	3.93	0.74	<0.001
	VNO_thick_	−0.65	−0.91	−0.91	−0.03	−0.24	0.19	0.01	0.902
	VNO_area_	−2.61	−2.30	−2.25	2.19	1.88	2.50	0.77	<0.001

**Figure 2:**
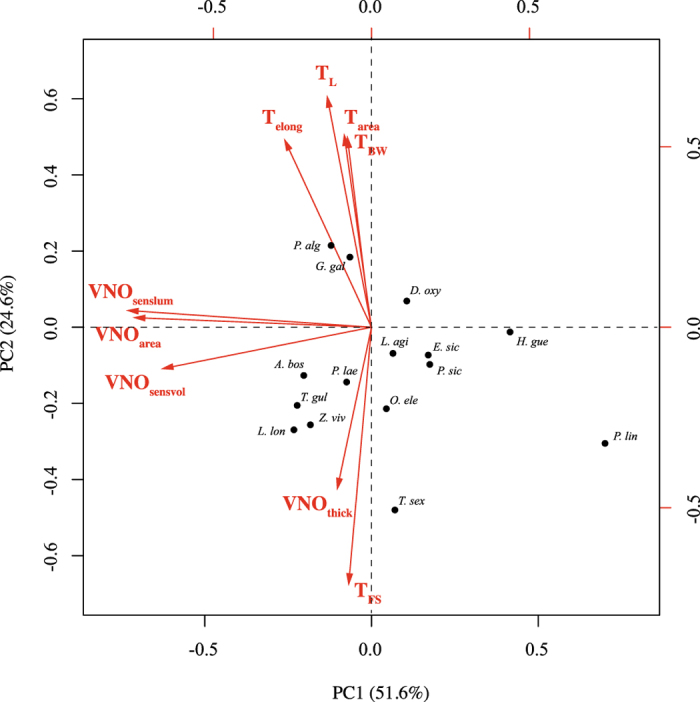
Biplot of the phylogenetic principal component analysis (pPCA) for the first two principal components (PCs) of 15 lacertid species. Red arrows indicate the PC loadings. The percentages of variance explained by the PCs are shown in the axis labels.

We performed a phylogenetic size correction for all lingual and VNO measures except T_FS_, T_elong_ and VNO_thick_. A phylogenetic principal component analysis with T_FS_, T_elong_ and VNO_thick_, and the phylogenetic residuals for T_L_, T_BW_, T_area_, VNO_senslum_, VNO_area_ and VNO_sensvol_ as input variables, yielded two component axes that jointly explained 76.2% of the variation (Fig. 2). The first component axis (51.6%) was strongly affected by VNO_senslum_ (loading = −0.73), VNO_area_ (−0.71) and VNO_sensvol_ (−0.63). *Pedioplanis lineoocellata* and *Holaspis guentheri* scored high on this first axis, indicating that these species have relatively small vomeronasal organs. *Latastia longicaudata*, *Tropidosaura gularis*, *Acanthodactyus boskianus* and *Zootoca vivipara* were species at the other extreme of this first axis. The second axis (24.6%) had highly positive loadings for T_L_ (+0.68), T_elong_ (+0.52), T_area_ (+0.51) and T_BW_ (+0.50), but highly negative loadings for T_FS_ (−0.68) and VNO_thick_ (−0.43). It separated species with relative long, broad tongues and a thin layer of VNO sensory epithelium (e.g. *Gallotia galloti* and *Psammodromus algirus*) from species with shorter, narrower and strongly forked tongues with a thick layer of sensory epithelium in their VNO (in particular, *Takydromus sexlineatus*).

The outcome of the pPCA suggested close co-variation among VNO-measures (except VNO_thick_) on the one hand and the lingual measures on the other hand (except T_FS_). The perpendicular orientation of the loadings suggests that most characteristics of the tongue evolved independently of VNO size (Fig. 2). However, T_FS_ and VNO_thick_ form an exception, as their loadings implied a tight association between them. Indeed, a pGLS analysis confirmed a significant positive relationship between T_FS_ and VNO_thick_ (*F*_1,14_ =5.20, slope = 0.31, *r* ^2^ = 0.29; *P* = 0.012).

### Relationship between the sending and the receiving system

**Figure 3:**
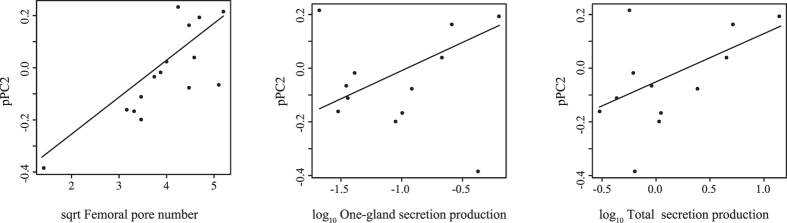
Graphs illustration the relationship (pGLS) between the morphology of the vomeronasal-lingual system (by PC2) of lacertid lizards and their investment in chemical signalling (as femoral pore number, one-gland secretion production and total secretion production).

To test for co-evolution between vomeronasal-lingual morphology and the signal producing system, we computed the scores of each species on the two pPCA axes and correlated these scores with femoral pore number, one-gland secretion production and total secretion production. No significant co-variation was found between scores on the first axis (VNO size), nor between the three measures of investment in signal production (all *P* > 0.41). However, scores on the second axis correlated strongly and positively with the number of pores (*P* < 0.001, slope = 0.14), one-gland secretion production (*P* = 0.02, slope = 0.21) and total secretion production (*P* = 0.02, slope = 0.18) suggesting that species with relatively small, low-forked tongues and VNOs with a thin layer of sensory epithelium tend to invest strongly in chemical signal production (Fig. 3; Table 3).

**Table 3:** Significance of the slope (i.e. different from 0) of the relationship between pPC1, pPC2 and the average number of femoral pores (FPN), one-gland secretion production (OSP), total secretion production (TSP), foraging variables PTM and MPM. Bold indicate statistical significance.

	FPN	OSP	TSP	PTM	MPM
pPC1	0.69	0.41	0.87	0.21	0.46
pPC2	**<0.001**	**0.02**	**0.02**	0.10	0.74

Slope for FPN = 0.14.

Slope for OSP = 0.21.

Slope for TSP = 0.18.

### Relationship between the receiving system and foraging behaviour

We found no significant correlation between pPCA scores (both axes) and indices of foraging behaviour (PTM, MPM) (all *P* > 0.10), thus providing no support for the idea that the size or shape of the tongue and VNO evolved as a function of foraging behaviour in lacertids (Table 3).

### Phylogenetic signal

**Figure 4:**
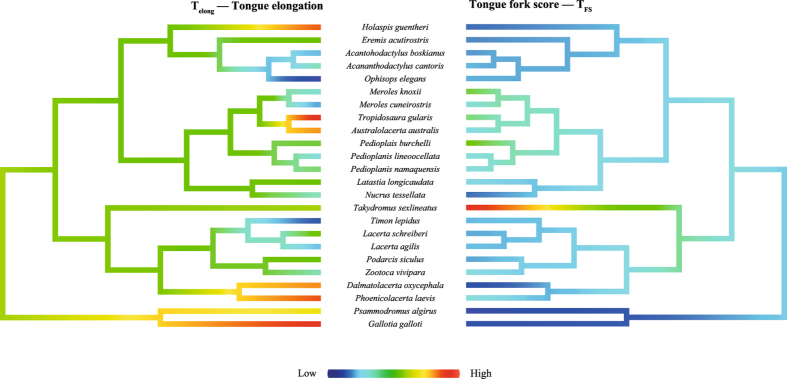
Ancestral character estimation of tongue elongation (T_elong_) and forkedness (T_FS_) along the branches and nodes of the tree for 24 lacertid lizard species. The illustration succeeds in visualizing the phylogenetic conservative character of both traits (Blomberg’s K > 1). Illustration made in R (function ‘contMap’, package ‘phytools’^97^).

The overall tongue and VNO morphology of lacertid lizards showed relatively weak phylogenetic signal (Blomberg’s K<0.8, *P* > 0.05; Pagel’s λ < 0.01, *P* > 0.05). The only exceptions were T_FS_ and T_elong_ that showed high λ values (both 0.99, *P* < 0.05) and K values over one (resp. 1.10 and 1.02, both *P* = 0.020), which implies that neighbouring lizard species tend to resemble each other more —in their degree of tongue elongation and forkedness—than expected under Brownian motion of evolution (Fig. 4).

## Discussion

### Variation in vomeronasal-lingual morphology

Squamates use their vomeronasal-lingual system to sample chemicals from the surroundings^47,48^. Several characteristics of the tongue and the VNO that are very likely coupled to efficiency of chemical sampling and processing are known to vary drastically among major squamate groups^1,2,5,17–20^. Our findings revealed marked differences in vomeronasal-lingual morphology at the species level. Still, the magnitude of interspecific variation is evidently smaller than reported among lizard families. To illustrate, a family-wide comparison by Cooper^3^ comprising ten lizard families shows that tongue elongation (T_elong_) ranges between 1.29 and 7.39 among families, whereas this interspecific study finds T_elong_ to range between 0.14 and 2.31 among lacertids; a factor of 26 difference in T_elong_ variance. The same trend is observed for tongue-fork score (T_FS_), which is established to range between 0.10 and 6.43 among lizard families^19^, but among lacertids, only ranges between 0.33 and 3.77 (this study), resulting in an among-family variance in T_FS_ that is approximately 14 times higher than the variance found among lacertids. Alas, the limited literature data on lizard VNO morphology prevents us from making meaningful comparisons on the degree of variation in VNO morphology at a low and high taxonomic level.

### Functional connection between sampler and sensor

Following the concept of symmorphosis, which postulates a quantitative match of design and functional demand within a functional system^28,49^, one might expect a strong link between the design of the sampling device (tongue) and the sensor (VNO), to allow ‘optimal’ vomerolfactiog. W.E. Cooper, Jr. was the first researcher to test this relationship in squamate reptiles. Based on an among-family comparison encompassing eleven squamate families (including Colubridae), Cooper^4,17^ argued for a close evolutionary relationship between the abundance of vomeronasal chemoreceptors and several aspects of lingual morphology, especially the degree of forking and elongation, and interpreted these results as an ‘optimization’ of the vomeronasal-lingual system. Nowadays, the findings of these studies might be considered as slightly equivocal, since Cooper’s analyses from the late nineteen-nineties combine highly distant-group comparisons with morphology-based phylogenies (which is fundamentally discordant with the current molecular squamate tree of Zheng & Wiens^50^).

Our study finds only limited support for co-evolution between the lingual and VNO system in lacertid lizards. Overall, our findings imply independent evolution of tongue form and VNO size. Yet, some specific characteristics do co-vary, as species with a high tongue-fork score (T_FS_) are also equipped with a thick layer of VNO sensory epithelium. Since bifid tongues may enhance tropotactic scent-trailing^19^ and since a thick sensory epithelium might increase the functionality, discriminatory ability, reliability and sensitivity of the VNO^47,48^, it is tempting to infer this co-variation between ‘sampler’ and ‘sensor’ as a functional ‘optimization’ of chemosensory design. Still, it is important to be cautious with the interpretation of these results. Firstly, while the thickness of VNO sensory epithelium serves as an excellent proxy for the chemosensory ability in mammals and amphibians because it is directly related to the number of sensory neurons in these animal groups^51,52^, this has not been investigated yet for lizards. The only validation for this proxy derives from qualitative anatomical descriptions reporting that the VNO sensory epithelium layer of vomerolfactory ‘specialists’ (snakes) is considerably thicker than those of vomerolfactory ‘generalists’ (iguanian lizards)^47,48,53^. Extra information on the proportion and total number of sensory neurons in the VNO epithelium, the thickness of the accessory olfactory axons, and the size of the accessory olfactory bulbs, and their connecting with the chemosensory behaviour of (lacertid) lizards, may provide clarification. Secondly, many questions about the true role of forked tongues in squamates still remain unanswered. While a high degree of bifurcation most likely enhances the ability for tropotactic scent-trailing^19^, it is uncertain whether lacertids are fundamentally capable of simultaneously comparing stimulus intensities on two sides of the body. The limited evidence on the function of forked tongues in locating and following trails solely derives from studies focussing on squamate groups with extreme bifid tongues, such as teiids, varanids and snakes^18,54–57^, and not lacertids. Besides, it can be questioned whether a tropotactic life-style is really profitable for the average lacertid lizard. Snakes and varanid lizards, for instance, often need to travel large distances (in the magnitude of several kilometres) in search for specific prey or mates^58–61^, and undoubtedly benefit from efficient chemosensory searching by tropotactic scent-trailing. Lacertids, however, might not necessarily profit from an investment in highly bifid tongues as they typically feed on the prey available in their relative small home ranges, and moreover,lacertids usually live in high-density populations^62–65^^.^ This might be an explanation of why the average degree of tongue bifurcation of lacertids (T_FS_) is 4.2 times smaller than those of varanids (following Schwenk^19^). Furthermore, although a highly forked tongue permits tropotaxis, it might not necessarily increases a lizard’s overall chemical sampling abilities. It is not unlikely to imagine that long, broad tongues with a large surface area increase the amount of chemicals that can be sampled in a single tongue-flick, and not slender, bifid tongues. However, whether such a tongue is also capable of efficiently delivering chemicals to the VNOs brings an additional question to the table. Besides, the fact that it is unclear which role tongue-flick rate exactly plays in the relationship between sampler and sensor, complicate things even more. Clearly, many aspects on the functional morphology of the lingual and VNO system demand extra attention in the near future.

### Drivers of rapid divergence in vomeronasal-lingual morphology

Foraging ecology is traditionally argued as the major force driving the variation in squamate chemosensory development on a high taxonomic level^5,17,19,21^. The most likely interpretation (following Cooper^5^) is that shifts in foraging mode drove changes in chemosensory behaviour and morphology. Because a proficient chemosensory system can also be used in chemical communication, active foraging species are also thought to invest more in chemical cues and signals. While the present study was unable to find an association between foraging activity and the chemosensory design of lacertids, it did establish a link between a lizards’ investment in chemical signalling and the morphology of their vomeronasal-lingual system. Our findings showed a relationship between tongue form and VNO sensory epithelium thickness of lacertids, and their investment in secretion production: species that carry many secretory glands and are able to produce large amounts of secretion, have on average a thin layer of VNO sensory epithelium and long, broad tongues that are only marginally forked (Fig. 3). This outcome advocates chemical communication with a role as potential player affecting chemosensory design in lacertids. However, based on the assumption that a maximally forked tongue and a thick layer of VNO sensory epithelium enhances chemical sampling and processing abilities, the direction in which secretion production relates with vomeronasal-lingual morphology was rather unexpected. There are at least three possible explanations for this result. First, a species that strongly invests in glandular secretions for chemical signalling could actually benefit from a long, broad tongue with a large surface area, as such a tongue-form might allow to sample more chemicals in a single tongue-flick than a bifid tongue can; this is solely based on the premise that a larger tongue surface area can sweep a larger area in air, independent of tongue-flick rate or kinematics^66,67^. Collecting a large amount of chemicals in a short period of time might permit a lizard to process (and react accurately on) the gathered information more easily and rapidly (e.g. for mate assessment). This might be more important than the need for directional mate trailing (thus a highly forked tongue). While this may be the case for lacertids, it is probably not true for squamates that are required to travel long distances to find mates. For instance, North American pitvipers, such as *Agkistrodon contortrix*, use chemical cues to search for prey and mates, and carry a bifid tongue to do so^68^. The males, however, have evolved an even more deeply forked tongue than their female conspecifics, as males are the mate-searching sex in these snakes and they need to compete with other males in their search for a proper mate^69^. A highly bifurcated tongue would therefore enhance tropotactic scent-trailing, hence the efficiency to find mates.

A second explanation can be ascribed to predation pressure. Lizard species that are subjected to a high predation pressure by chemically-oriented hunters (such as snakes), might benefit from (1) reducing the amount of glandular secretion they deposit in order to lower the (potential) detrimental effect of predatory eavesdropping, (2) carrying a highly bifid tongue to accurately pinpoint the direction of danger using tropotaxis. Some snakes are known to react strongly on chemical cues originating from the femoral gland secretions of lacertids (e.g. *Coronella austriaca* ^70^), Also, lacertid lizards are able to ascertain the presence of snakes by scent, and some even can discriminate chemical cues of snakes that are saurophagous from those which are non-saurophagous (e.g. *Zootoca vivipara* ^71^ and *Podarcis muralis* ^37^).

Thirdly, functional and/or genetic factors might constraint the co-evolution of certain tongue or VNO characteristics. It is not unlikely that, for example, tongue elongation and tongue forkedness in lacertids are restrained to co-develop. Interestingly, our study revealed strong phylogenetic signal in both these tongue variables. Species belonging to the Gallotiinae clade for instance, such as *Psammodromus algirus* and *Gallotia galloti* ^72^, carried highly elongated tongues with small tines. The two species of the genus *Acanthodactylus* had only slightly forked tongues, whereas the SouthAfrican lacertids *Tropidosaura gularis* and *Australolacerta australis*, both belonging to the Eremiadini clade^73^, possessed highly elongated tongues. Whether the phylogenetic conservative character of these traits is constraining the adaptive evolution of tongue-form remains unclear.

Although plausible, the above-mentioned explanations still remain speculative. Without doubt, more extensive research, including other proxies for signalling investment, such as tongue-flick rate and kinematics, and focussing on the causality of the relationship between chemosensory design and signalling investment, is a necessity.

While the level of chemical signalling investment has potential to drive variation in the chemosensory system of lacertids, other players, such as foraging behaviour, might be affecting the observed variation too. Lacertids are generally categorized in the group of ‘active’ foragers, rather than ‘ambush’ foragers^74^, still, species are known to differ in their quantitative level of foraging activity^42,43,75^. Our study was, however, unable to find a statistically significant association between foraging activity and vomeronasal-lingual morphology in lacertids. On a high taxonomic level, Schwenk^19^ was able to establish a relationship between the degree of tongue forkedness and foraging behaviour. However, these findings should be interpreted with a certain note of caution as they derive from comparisons among (merely) nine distant lizard families, while ignoring the effects of shared-ancestry. The fact that this link was undetectable on a within-family level suggests that foraging behaviour is less important in driving variation in tongue and VNO morphology in the evolutionary history of lacertid lizards. Another possibility is that the interspecific variation in lacertid foraging activity and vomeronasal-lingual morphology is too subtle to be picked up by our analyses. Although there is considerable interspecific variation in the percentage of time moving in lacertids^75^, the variance is still approximately 1.5 times smaller than observed among lizard families^76^. It is also likely that foraging ecology rather affects chemosensory behaviour than vomeronasal-lingual morphology. Indeed, a broad-scale comparative study encompassing nearly 100 squamate species shows that highly active foragers tongue-flick at a higher rate than less active foragers^29^. Scholars should be encouraged to apply an integrative approach whilst investigating species’ relative reliance on chemicals to find prey, to detect predators, and in an intraspecific social context.

## Material and Methods

### Study animals

We used preserved specimens representing 26 species of Lacertidae that were available in the Ellerman Collection of the University of Stellenbosch (SouthAfrica), the Zoological Museum of Tel Aviv University (Israel), the Laboratory of Functional Morphology at the University of Antwerp (Belgium), and in private collections of A. Herrel (Muséum National d’Histoire Naturelle in Paris, France) and J. Martín (Museo Nacional de Ciencias Naturales in Madrid, Spain). Specimens were decapitated (at approximately 5 mm caudal to the posterior extremity of the parietal scale), and their heads were placed in a staining solution of 5% phosphomolybdic acid (PMA; Sigma-Aldrich, St Louis, MO, USA) and 70% EtOH for a minimum period of 14 days (adapted from^77,78^), while the rest of the lizard body was retained in 70% EtOH. PMA is similar to the phosphotungstic acid (PTA) stain, and has proven equally suitable or better for staining animal soft tissues^79,80^. After staining, we recorded for each individual lizard specimen the length, width and height of the head (precision = 0.01 mm). Head length (H_L_) was measured from the posterior extremity of the parietal scale to the tip of the snout. Head width (H_W_) was the largest distance measured between the temporal scales, and head height (H_H_) was the maximum distance measured between the base of the mandible and the parietal surface. Only adult male lizards were included in this study.

### VNO morphology

We used micro-computed tomography (micro-CT) to acquire reconstructed cross sectional image data of the VNOs of lacertid lizards. Micro-CT scanning was performed with a high resolution SkyScan 1172 X-ray scanner (Bruker Micro-CT, Kontich, Belgium) located at the Free University of Brussels (VUB, Belgium). After staining, lizard heads were removed from the solution and firmly mounted on a metal disc using adhesive wax (orthodontic tray wax, Kerr, Bioggio, Switserland), and placed in a closed-off custom-made Plexiglass case to avoid drying artefacts (in ref.^81^). Over a range of 180 degrees and using an aluminium-copper filter, 360 X-ray shadow images were taken every 0.5 degrees, with an averaging of 4 frames for noise reduction. In order to arrive at optimal contrast of the X-ray attenuation by the sample, the settings for voltage, current and exposure time were manually optimized per sample according to the advanced imaging protocols of the manufacturer (i.e. Bruker). The scanning time was approximately 1 hour (per sample), which ultimately produced reconstructed images of 5.40 μm mean voxel size.

**Figure 5:**
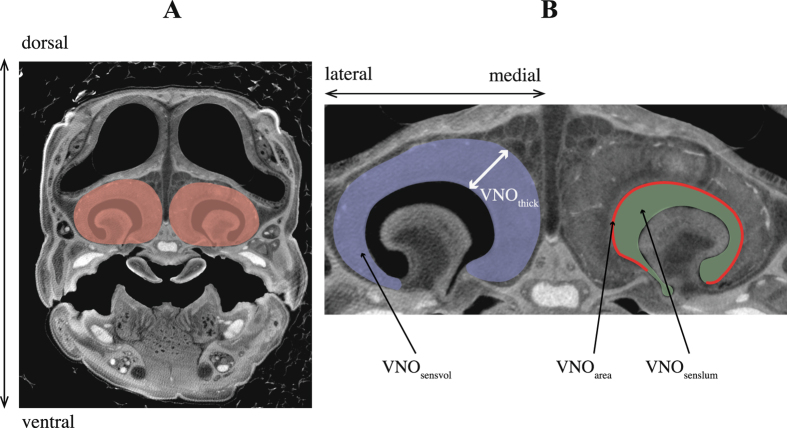
An example of reconstructed micro-CT images of a lacertid lizard head (*Takydromus sexlineatus*) highlighting the vomeronasal organs (VNOs). (**A**) transverse section of the complete head, with the VNOs in red; (**B**) transvers section focussing on the VNOs, with additional annotations on the measures used in this study. Abbreviations: VNO_sensvol_ = volume of VNO sensory epithelium (blue), VNO_senslum_ = volume of VNO lumen (green), VNO_thick_ = thickness of VNO sensory epithelium (white line with arrows), VNO_area_ = surface area of VNO sensory epithelium (red line).

Image segmentation, which is the outlining of relevant structures in the cross sectional image data, was performed with the 3D image processing software package Amira 5.4.3 (64-bit version; VSG systems, Mérignac, France). Using the reconstructed slice images, the vomeronasal sensory epithelium and lumen of the left and right VNO were semi-automatically segmented for each specimen based on grey scale values, and their volumes were calculated (VNO_sensvol_ and VNO_senslum_, respectively). Additionally, the surface area of the sensory epithelium that makes direct contact with the VNO lumen was quantified (VNO_area_). Lastly, we estimated the sensory epithelium thickness (VNO_thick_). To do so, we measured the width of the epithelium on five standardized anatomical locations on the transverse image data and calculated the mean; starting from the first (rostral) section image where the VNO fenestrae were clearly visible, epithelia width was measured every 5 images, for 5 times. For each species, left and right VNO morphology was almost identical for all dimensions measured, and therefore the calculated means of the left and right VNO variables were used in all further analyses. An example of a cross-section micro-CT image of a lizard head, with annotations on the VNO measures, is shown in Fig. 5. We assume that the VNO sensory thickness, volume and surface area of lizards, reflects the functional importance of their chemosensory system, and their overall reliance on chemoreception^5,17,53,74^. We further assume that species with a relatively thicker sensory epithelium, larger VNO volume and larger VNO surface area have a higher chemosensory sensitivity, discrimination level and reliability. All segmentations and calculations in Amira were performed by a single operator, and all settings were standardized for all scans.

In total, we scanned and analysed the scans of 17 different lacertid species. For 16 species, only 1 specimen was scanned. To check and account for the degree of intraspecific variation in VNO morphology, we scanned seven specimens of the species *Podarcis peloponnesiacus*. After normalisation for head length, the observed among-species variation (as SD) was on average 3.3 times larger than the within-species variation. This result validates the reliability of interspecific VNO comparisons in this study (see electronic supplementary material).

### Tongue morphology

**Figure 6:**
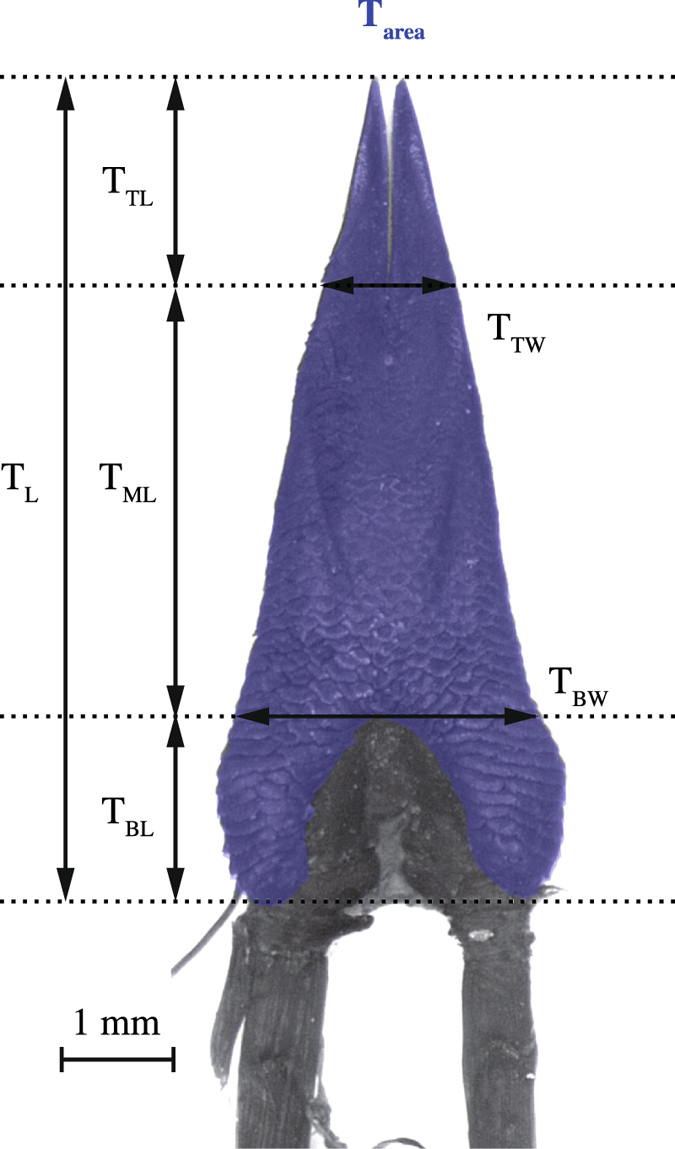
Photograph of a lacertid lizard’s tongue (dorsal view) with annotations on the morphological variables considered in this study. The surface area of the tongue is coloured. The tongue given for reference is from the species *Acanthodactylus cantoris*. Abbreviations: T_BL_ = tongue base length; T_BW_ = tongue base width; T_ML_ = tongue mid length; T_TL_ = tongue tip length; T_TW_ = tongue tip width; T_L_ = tongue length; T_area_ = tongue surface area.

After micro-CT scanning, we dissected the tongues of 15 out of the 17 scanned species, plus the tongues of nine additional species. Subsequently, we photographed the dorsal surface of the tongues of all 24 species through a stereomicroscope (LeicaM165C). A thin cover glass gently pressed on the tongue’s surface area aided in making standardized images. Based on those images, several morphological variables of the tongue were estimated using the ImageJ software (Abràmoff *et al*. 2004): base length and width (T_BL_ and T_BW_), tip length and width (T_TL_ and T_TW_), mid length (T_ML_), total length (T_L_), and tongue surface area (T_area_) (see Fig. 6). An additional variable, scoring the degree of lingual forking, was calculated by dividing the tip length by the tip width, and labelled T_FS_ (‘tongue fork score’, following^4,18^). Lastly, ‘tongue elongation’ (T_elong_), was estimated as tongue-mid length divided by tongue-base width (as in ref.^3^).

Because we took three photographs of each tongue, we were able to assess the repeatability of the method used to analyse tongue morphology. Following procedures outlined by Lessells & Boag^82^, we obtained a repeatability of over 98% for all variables. In subsequent analyses, we used the mean of the three measurements obtained for each species.

### Data on chemical signalling system and foraging behaviour

To test whether the vomeronasal-lingual system morphology of lacertids co-varies with their investment in chemical signalling and/or foraging ecology, we collected data on the species’ chemical signal quantity, and foraging behaviour.

**Figure 7:**
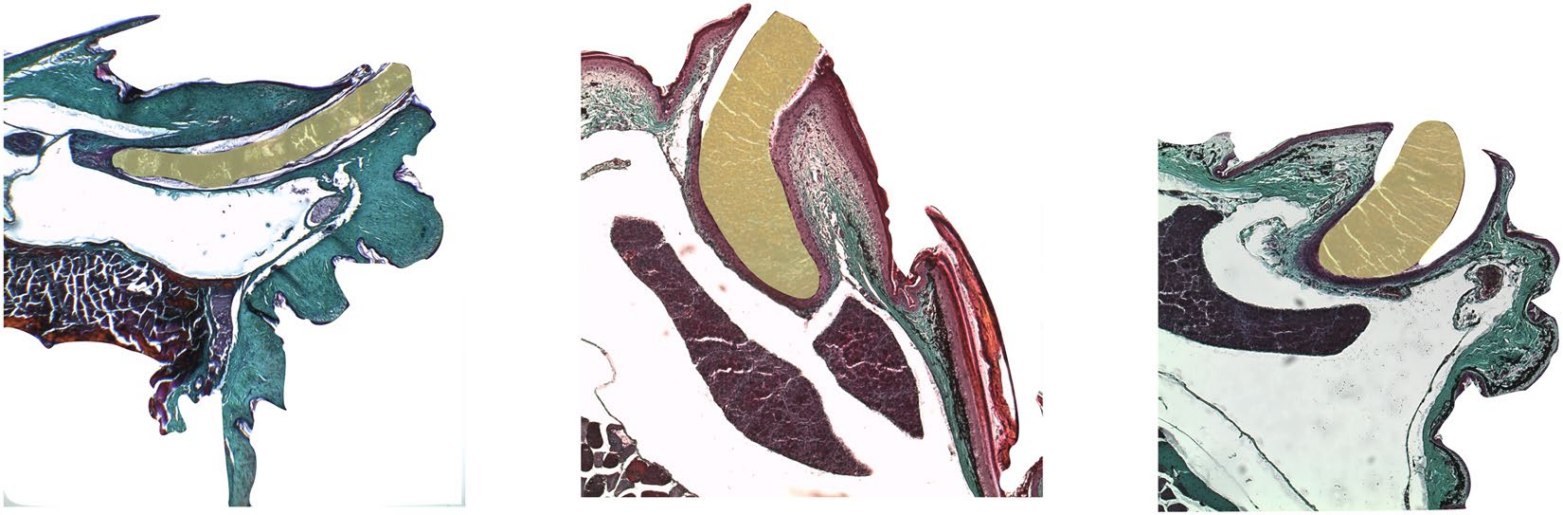
Photographs of transverse sections through the femoral glands of three lacertid lizards (from left to right: *Timon lepidus*, *Tropidosaura gularis*, *Australolacerta australis*). The femoral gland secretions or ‘ secretion plugs’ are coloured yellow.

In lizards, the leading source of chemical signals involved in chemical communication is considered to be the femoral gland secretion (reviewed by refs^27,45^). The femoral glands, located in the dermis of the inner thighs, produce a waxy lipophilic-rich secretion that finds its way to the external world through pore-bearing scales (i.e. femoral pores). The number of femoral glands/pores is known to vary greatly among lacertid species^46^. We selected secretion gland production as a proxy for a species’ investment in chemical signalling, and based ourselves on two features: femoral pore number and secretion quantity. Data on the former was collected by counting the number of pores on the left hind limb of each lizard, and information on secretion quantity was gathered from histological sections of species’ femoral glands. A small fragment of the femoral gland patch (5 mm²) was excised from the hind leg of one adult male per species and prepared for paraffin histology using standard methodology^83^. Serial transverse sections (4–8 *µ*m) were obtained from the femoral patch and stained with Masson–Goldner trichrome^84^. Thereafter, we estimated the (one-gland) secretion production of each species by digitizing (ImageJ^85^) the circumference of the ‘secretion plug’ on images obtained with the stereomicroscope (Fig. 7). Finally, as a proxy for the total secretion production of a species, we multiplied the number of pores (thus glands) with the size of the secretion plug. All preserved specimens were captured during their reproductive season in which gland activity is maximal^86–88^.

Two widely established numerical parameters for describing lizard foraging behaviour activity are (1) the number of moves per minute (MPM), and (2) the percent of time spent moving (PTM)^76,89^. For 13 species included in this study, we were able to extract average MPM and PTM scores from the literature^75,90–93^.

### Phylogenetic comparative analyses

Because species cannot be treated as independent data points, we performed all analyses within a phylogenetic context, hence accounting for shared-ancestry. We based our lacertid phylogeny on the one used by Baeckens *et al*.^46^, which is a Bayesian phylogeny constructed by the use of three mitochondrial and two nuclear gene regions. We obtained our estimate of the phylogeny by pruning Baeckens’ tree to include only the 26 species of this study. Prior to analysis, all morphological variables were log_10_-transformed and count variables were square-root-transformed to meet the assumptions of normality (Shapiro-Wilk’s test with W ≥ 0.95). Thereafter, we assessed the relationships among head size (as H_L_) and, tongue and VNO morphology using phylogenetic generalised least square regressions (pGLS; function ‘pgls’, package ‘caper’^94^). Because we were primarily interested in shape variation, we performed a phylogenetic size-correction for all lingual and VNO measures that were affected by head size, and used the phylogenetic residuals to index shape variation in subsequent analyses (function ‘phyl.resid’, package ‘phytools’^95^). We used a phylogenetic principal component analysis (pPCA; method ‘lambda’) on tongue and VNO variables to examine co-variation between the lingual and vomeronasal system. To reduce the number of input tongue variables in the pPCA analysis, we only included one variable for the length of the tongue (i.e. T_L_), and one for the width (i.e. T_BW_), hence excluding T_BL_, T_ML_, T_TL_, T_BW_ and T_TW_. Relationships between the vomeronasal-lingual system and the investment in chemical signalling and foraging behaviour were tested using pGLS regressions based on species’ scores obtained from the pPCA. Chemical signalling variables were not size-corrected as none of them were affected by head size (pGLS, all *P* > 0.14). Lastly, the phylogenetic signal for all tongue and VNO variables was estimated using Pagel’s λ and Blomberg’s K (function ‘phylosignal’, nsim = 10000, package ‘phytools’^96^).

## Electronic supplementary material

Supplementary Information
