# Author Correction: Evolutionary morphology of the lizard chemosensory system

**DOI:** 10.1038/s41598-017-17185-5

**Published:** 2017-12-11

**Authors:** Simon Baeckens, Anthony Herrel, Chris Broeckhoven, Menelia Vasilopoulou-Kampitsi, Katleen Huyghe, Jana Goyens, Raoul Van Damme

**Affiliations:** 1 Laboratory of Functional Morphology, Department of Biology, University of Antwerp, Universiteitsplein 1, 2610 Wilrijk, Belgium; 2 UMR7179, CNRS/MNHN, 75005 Paris, France; 3 Department of Botany & Zoology, Stellenbosch University, Private Bag X1, Matieland, 7602 Stellenbosch, South Africa; 4 Present Address: Department of Organismic and Evolutionary Biology, Harvard University, Cambridge Massachusetts, USA

*Scientific Reports* **7**:10141; doi:10.1038/s41598-017-09415-7; Article published online 04 September 2017

This Article contains errors in Figure 4, where “Eremis acutirostris” should read “Eremias acutirostris”, “Acananthodactylus cantoris” should read “Acanthodactylus cantoris”, and “Pedioplais burchelli” should read “Pedioplanis burchelli”. The correct Figure 4 appears below as Figure 1.

**Figure 1:**
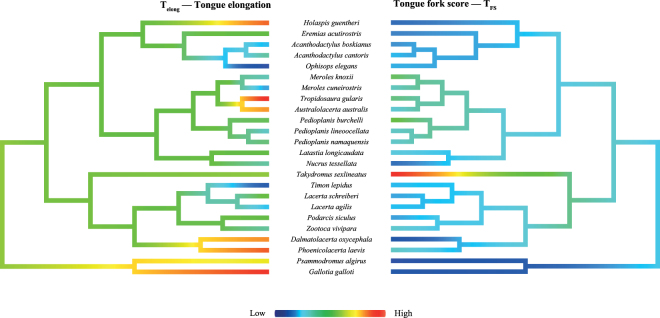
.

