# Supplementary material for: Evolutionary morphology of the lizard chemosensory system
Source: Sci Rep. 2017 Sep 4;7:10141. doi: 10.1038/s41598-017-09415-7 (PMC5583331; doi:10.1038/s41598-017-09415-7)
Supplement: Supplementary file 1 — Supplementary Information [file 41598_2017_9415_MOESM1_ESM.pdf]

Evolutionary morphology of the lizard chemosensory system

Simon Baeckens[1,*] , Anthony Herrel[2], Chris Broeckhoven[3], Menelia Vasilopoulou-Kampitsi[1], Katleen Huyghe[1], Jana Goyens[1] and Raoul Van Damme[1]

[1] Laboratory of Functional Morphology, Department of Biology, University of Antwerp, Universiteitsplein 1, 2610 Wilrijk, Belgium

[2] UMR7179, CNRS/MNHN, 75005 Paris, France

[3] Department of Botany & Zoology, Stellenbosch University, Private Bag X1, Matieland 7602, Stellenbosch, South Africa

* Corresponding author.

E-mail address: simon.baeckens@uantwerp.be

Short running title:

Evolution of the lizard chemosensory system

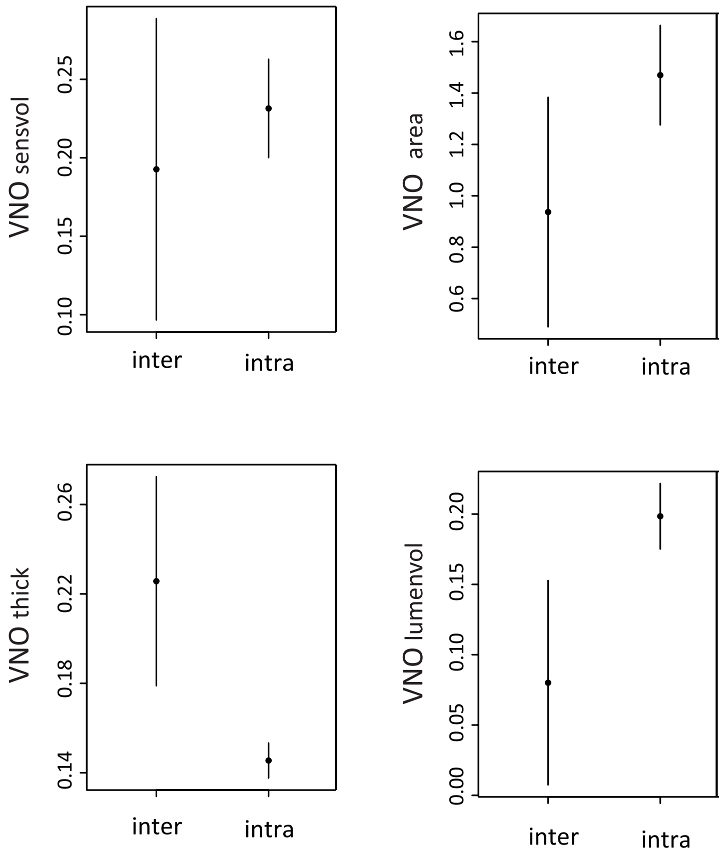

Figure Supplementary Material — Graphs showing the interspecific and intraspecific mean values for the different vomeronasal organ (VNO) measurements used in this study. Means are calculated and presented for 16 species (interspecific; ‘inter’) and for 7 individuals of one species, *Podarcis peloponnesiacus* (intraspecific; ‘intra’). Error bars are standard deviations (SDs) and show that among-species variation is much larger than within-species variation.
